# Unexpected Therapeutic Implications: The Abscopal Effect in the Management of Hepatocellular Carcinoma

**DOI:** 10.3390/cancers18030408

**Published:** 2026-01-28

**Authors:** Lucia Cerrito, Maria Pallozzi, Ilaria Urbani, Sebastiano Archilei, Sara Miliani, Elisabetta Creta, Leonardo Stella, Antonio Gasbarrini, Francesca Romana Ponziani

**Affiliations:** 1 Liver Unit, CEMAD Centro Malattie Apparato Digerente, Department of Internal Medicine and Gastroenterology, Fondazione Policlinico Universitario A. Gemelli IRCCS, 00168 Rome, Italy; 2 Department of Life Sciences, Health and Health Professions, Link Campus University, 00165 Rome, Italy; 3 Department of Translational Medicine and Surgery, Catholic University of the Sacred Heart, 00168 Rome, Italy

**Keywords:** hepatocellular carcinoma, abscopal effect, systemic therapies, radiotherapy

## Abstract

Hepatocellular carcinoma (HCC) is the sixth most diagnosed cancer worldwide and represents the second cause of cancer-related death worldwide, according to the estimates by Global Cancer Observatory in 2020. Its prognosis is strictly associated with neoplastic stage and liver function. Several treatments are involved in the management of HCC, according to its stage and the patients’ performance status. The abscopal effect is a particular phenomenon taking place in locally advanced or in metastatic cancer, as a positive off-target result of ionizing radiation at a certain distance from the irradiated zone determining a systemic effect due to the increase in tumor immune response, and resulting in the shrinkage of neoplastic lesions with a subsequent increase in tumor prognosis. If future studies will allow one to master its etiological mechanisms and deliberately trigger them, it could represent a notable weapon in the treatment of hepatocellular carcinoma, particularly at advanced stages, in which sometimes it is able to positively modify the patients’ prognosis. The aim of this review is to evaluate the literature available on this intriguing topic and the characteristics of the mechanisms that originate the abscopal effect, the treatments that can elicit this phenomenon, and the possible relevant therapeutic implications.

## Simple Summary

Hepatocellular carcinoma is estimated to be the second cause of cancer-related death worldwide and its prognosis is strictly related to both neoplastic stage and liver function. Several treatments are involved in the management of hepatocellular carcinoma. The abscopal effect is a particular phenomenon taking place in locally advanced or metastatic cancer as a positive off-target result of ionizing radiation at a certain distance from the irradiated zone, determining a systemic effect due to the increase in tumor immune response and resulting in the shrinkage of neoplastic lesions with a subsequent increase in tumor prognosis. The progressive understanding of the mechanisms underlying the abscopal effect could lead to important progress in terms of therapeutic solutions for the hepatocellular carcinoma.

## 1. Introduction

Hepatocellular carcinoma (HCC) represents the sixth most diagnosed cancer worldwide in 2020 and is the third most frequent cause of cancer-related death worldwide, according to the estimates by Global Cancer Observatory. Its prognosis is closely associated with the neoplastic stage, patient’s clinical conditions, and liver function, ranging from 70% at 5 years from the diagnosis in the early stages to 20% in advanced stage [1]. There is a growing concern about the incidence and mortality of primary liver cancer: H. Rumgay et al. analyzed the GLOBOCAN 2020 database, showing approximately 900,000 new diagnoses/year with around 830,000 deaths. Prospective data show a 55% increase in new diagnoses and deaths in future years [2], especially in developed countries, where the main causes of HCC are alcohol use disorder, followed by viral hepatitis (with a progressively decreasing incidence), and metabolic dysfunction-associated steatotic liver disease (MASLD) that is progressively surpassing the other aetiologies [3,4].

## 2. Therapeutic Paths for Advanced-Stage HCC

Several treatments are involved in HCC management, according to the HCC stage and patients’ performance status: liver transplantation, curative resection, radiofrequency ablation, trans arterial chemoembolization, radioembolization, systemic therapy, radiotherapy [5]. Particularly, during the last few years, the systemic treatments for unresectable or advanced HCC have undergone profound changes due to intense and continuous research in this field. The scenario has shifted from first-line therapies based on the use of tyrosine kinase inhibitors (TKIs), such as sorafenib and lenvatinib, to first-lines based on immunotherapy due to the advent of immune checkpoint inhibitors (ICIs) even combined with other agents (e.g., anti-vascular endothelial growth factor A, anti-VEGF A such as bevacizumab) in order to improve the therapeutic effects of both pharmacological categories [5]. Immunotherapy-based combinations of treatment strategies have significantly increased patients’ survival: the main therapeutic strategies are represented by atezolizumab (a programmed cell death ligand 1 antibody-PD-L1) and bevacizumab (anti-VEGF), or the double immunotherapy based on tremelimumab (anti-CTLA-4 “Cytotoxic T-Lymphocyte-Associated protein 4”) and durvalumab (anti-PD-L1), as well as camrelizumab (anti-programmed cell death 1 “PD1”) and rivoceranib (anti-VEGF) that are diffused in Asian countries [5,6,7,8]. Even if they granted promising results demonstrated by several clinical trials and led to a revolution in HCC management, the objective response rate with first-line systemic therapy is only around 30% due to the extreme complexity of the neoplastic microenvironment and the absence of approved biomarkers that could reliably predict the response to such treatments [9,10].

Due to the introduction in clinical practice of these therapies, the activity of the immune response in achieving HCC disease control is progressively gaining high scientific interest and a growing relevance in the management of neoplastic diseases both in terms of pathogenesis and of disease treatment. In this multifaceted scenario, the abscopal effect, a rare phenomenon determined by immune mechanisms, may achieve a key role, even if currently the specific biological mechanisms underlying it are still under investigation [11].

Moreover, these treatments are often limited in efficacy and safety for patients with large tumor burden; for this reason, the researchers attempted to combine systemic therapies with locoregional treatments to reach a best disease control [12].

In some cases, the combination of immune checkpoint inhibitors with local treatments may induce synergistic therapeutic effects. The Society of Interventional Radiology (SIR) recently stated the curative potential of the combination of radiotherapy and ICIs for patients with intermediate or advanced HCC: due to the absence of detailed information regarding the real clinical impact and its management, further research is mandatory to understand the ideal sequencing of these combined treatments [13].

Radiation therapy has multiple roles in the oncological area: it is used to reduce the risk of recurrence after surgery, as a curative treatment in some localized cancers (e.g., early-stage prostate cancer), or as a palliative modality in metastatic cancer to reduce tumor bulk and relieve symptoms such as pain or mechanical compression of other organs [14,15].

The combination of radiotherapy and immunotherapy can elicit two different biological phenomena: the abscopal effect and the bystander effect. While the bystander effect is a local phenomenon, an intercellular signaling that takes place in non-irradiated cells near irradiated ones, the abscopal effect is a systemic, immune-mediated response leading to regression in distant tumor sites. The bystander effect is mainly local and does not require the activation of the immune system in distant sites. Both are relevant in cancer therapy, but the abscopal effect is of particular interest in hepatic carcinoma when integrating radiotherapy with immunotherapy [16,17].

## 3. The Abscopal Effect

The abscopal effect is a rare immune-mediated phenomenon observed principally in advanced cancer and is characterized by a regression of the tumor out of the area treated with radiotherapy, resulting in a systemic effect as a consequence of a local treatment. Its reports increased after the introduction of the combination of stereotactic body radiotherapy (SBRT) with ICIs [18]. Unfortunately, the abscopal effect still represents a dilemma due to the rarity of its frequency and the exiguous number of clinical cases reported in scientific literature; in this review, due to the substantial absence of well-structured randomized clinical trials because of the rare and unpredictable occurrence of the abscopal effect, we searched the literature through Pubmed and analyzed the principal papers regarding this phenomenon, consisting of case reports, small retrospective studies, and preclinical models. Unfortunately, the lack of more systematic studies impairs the deepening of the current knowledge regarding this theme and reduces the possibility to generalize the concept on a wider scale of patients.

The abscopal effect is determined by a systemic anti-tumor response described in several types of tumors (lung cancer, renal cell carcinoma, melanoma, lymphoma, HCC) after local treatments (e.g., conventional and stereotactic radiotherapy, thermal ablation), especially when combined with immunotherapy [11,19].

The term abscopal derives from the Latin expression “ab scopus” that means “away from the target” or “out of field” and refers to a phenomenon that has been considered quite rare in HCC so far and in which the combination of immunotherapy (particularly, inhibitors of PD-1 or PD-L1) with localized treatments (more frequently observed in HCC at advanced stages, particularly if metastatic) is able to interfere with HCC microenvironment, thus determining the reduction in tumor burden, not only of the targeted lesion, but also in sites that are far from the irradiated zones, boosting the activation of a systemic immune response with an anti-neoplastic activity [15,20].

It was first described by Mole in 1953, but it has been documented occasionally and, therefore, has not been considered a therapeutic target for a long time. In fact, it is rare with radiotherapy as single treatment, but a recently increasing number of abscopal responses have been reported with the routine use of ICIs, demonstrating that radiotherapy combined with immunotherapy has a synergistic effect on both irradiated and non-irradiated tumors. Currently, some clinical trials investigating the use of radiotherapy with ICIs are reported, although the optimal combinations to generate an abscopal effect are unclear [18,21,22].

It is also crucial to understand and deepen the knowledge about the mechanisms of this phenomenon in order to increase the potential beneficial effects [15].

The frequency of the abscopal effect increases in the case of combined treatments based on the association of ICIs [anti-programmed death-1 (PD-1) and anti-programmed death ligand-1 (PD-L1)] with radiotherapy [15]. The abscopal effect is more evident in subjects who are not candidate to “radical” locoregional treatments and who are administered, for this reason, high-dose radiotherapy in a hypo fractionated way, as in the case of SBRT or proton beam therapy, in combination ICIs [23].

The principal obstacle to such a remarkable therapeutic tool is represented by the immune-tolerance of the neoplastic microenvironment, that may hinder the occurrence of a clinically efficient abscopal effect [15].

## 4. The Mechanisms Underlying the Abscopal Effect

### 4.1. The Molecular Mechanisms of the Abscopal Effect

The neoplastic tissue upregulates PD-L1, produces TGF-β, and recruits suppressive immune cells (myeloid-derived suppressor cells and regulatory T cells), limiting the activated T cell response against the tumor and granting an immune-tolerance that preserves it from the attacks of the immune system.

**Figure 1:**
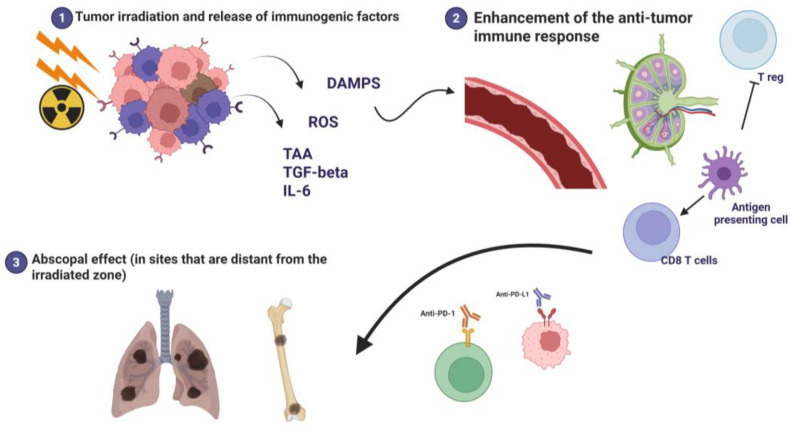
Radiotherapy and immune checkpoint inhibitors have a synergistic action in triggering the abscopal effect. Created with BioRender.com.

High doses of locally applied radiotherapy determine cell death mediated by immunogenic mechanisms: the ionizing radiation damages cancer cells through direct disruption of DNA by high-energy photons and the generation of reactive oxygen species (ROS). Radiation-induced damage exerts a potent immunomodulatory effect and causes the release of pro-inflammatory signals, damage-associated molecular patterns (DAMPs), cytokines (Transforming Growth Factor-beta “TGF-β”, interleukin-6 “IL-6”), and tumor-associated neoantigens (TAAs) that activate the dendritic cells (DCs) that capture the released antigens and, stimulated by danger signals, mature and migrate towards the lymph nodes. In the lymph nodes, DCs present antigens to cytotoxic T lymphocytes (CD8+), thus determining their priming, activating and instructing them to specifically recognize tumor cells. Activated T cells enter blood flow and migrate throughout the body, recognizing and attacking tumor cells that express the same antigens, including distant, non-irradiated metastatic lesions (Figure 1).

The DNA damage-induced cellular response alters the immunogenicity of irradiated tumor cells and causes a reshaping of the inflammatory microenvironment that underlies the abscopal effect [15,24]. The association of radiotherapy and ICIs (more often anti-PD-1 or anti-PD-L1) amplifies this reaction, allowing the downgrading of distant lesions due to the overtaking of the mechanisms of immune-tolerance and resistance established by the tumor and increasing the systemic response by cytotoxic T cells against non-irradiated neoplastic sites that did not receive irradiation [25,26].

Furthermore, studies of the gut–liver axis have expanded our understanding of the pathophysiology of various liver diseases and the mechanisms underlying the regulation of the effectiveness of therapies: the specific role of the gut microbiome in the regulation of the response to radiotherapy and ICIs has yet to be investigated. The prospective study by Li et al. underlines that radiotherapy on HCC can determine a modulation of patients’ intestinal microbiome, leading to an increase in HCC sensitization to radiation; primary resistance to radiotherapy may be attributed to the dysbiosis of gut microbiome, resulting in an impaired antigen-presentation process and the inhibition of the function of effector T cells. This result is obtained through the pathway involving the stimulator of interferon genes (STING). Even cyclic dimeric adenosine monophosphate (AMP) has been identified as a possible messenger of microbiome bacteria, acting as STING agonist and gaining further importance as a possible element for the modulation and prediction of response to radiotherapy in HCC [27].

### 4.2. Who Can Benefit from the Abscopal Effect?

It is certain that pre–existing liver diseases (chronic hepatitis B and C, cirrhosis, MASLD) notably shape the HCC immune microenvironment and the response to the treatments that should induce the abscopal effect. Basically, the HCC is able to modulate the immune response leading to an immune–tolerant microenvironment (increased activation of regulatory T cells and of suppressive macrophages that can delete cytotoxic CD8+ T cells and weaken the systemic immune responses) and several differences in the profile of immune activation have been detected according to the different etiologies [28,29,30,31]. Even chronic viral hepatitis and liver cirrhosis are involved in the genesis of neoplastic immune-tolerance through the increase in immunosuppressive cellular groups (myeloid-derived suppressor cells and regulatory T cells), the alteration of the CD4/CD8 T cell ratio, and the release of specific cytokines [10,30,31,32].

This effect is rarely observed in the case of HCCs, in patients treated with locoregional therapies alone, and it is less common in subjects affected by non-viral liver diseases (e.g., MASLD) characterized by poorer response rates to immunotherapy [33,34]. In fact, patients affected by non-alcoholic steatohepatitis or HCCs with an underlying activated Wnt/β-catenin signaling may have “cold” (non-inflamed) tumors determining intrinsic resistance mechanisms, resulting in a reduced response rates to ICIs and less chance of presenting an abscopal effect [35,36,37]; in more detail, MASLD and Metabolic Dysfunction-Associated Steatohepatitis (MASH) are characterized by dysfunctional CD4+ T cells activity and an upregulation of indoleamine 2,3-dioxygenase that diminishes the response of HCC to immunotherapy [29,38]. Conversely, some case reports underline that in patients with HCC developed on viral-related liver disease it is easier to observe an abscopal effect due to their distinct neoplastic microenvironment forged by viral inflammation [20,39].

Undoubtedly, the population gaining much benefit from the abscopal effect is represented by those in which the synergistic action of local radiotherapy with ICIs is clinically more evident: patients with advanced or metastatic HCC, especially with heavier tumor burdens, metastases located in extrahepatic districts, or with portal vein tumor thrombosis [20,40].

Patients treated with combination regimens of immunotherapy (such as anti-PD-1 or anti-PD-L1 plus anti-VEGF, anti-PD-1, or anti-PD-L1 plus anti-CTLA-4) are more likely to develop abscopal effect with higher response rates [40].

The majority of clinical trials, taking into consideration the abscopal effect, enrolled only Child–Pugh score A patients (with well-compensated liver function), thus limiting the knowledge about subjects with more advanced liver cirrhosis (Child–Pugh score B or C) and reducing the possibility to generalize some results to the whole population of patients with HCC [32].

The occurrence of the abscopal effect is unpredictable so far and the absence of specific validated biomarkers, linked to the possibility to predict its response, hampers the possibility to shape HCC response to combined treatments.

## 5. The Treatments for HCC Associated with Abscopal Effect

### 5.1. Ablative Treatments

Thermal ablative therapies are preferentially used as a treatment for HCC with localized disease: it has been observed that this kind of treatment can trigger the abscopal effect by anti-neoplastic immune response following the local destruction of tumor cells. Microwave ablation (MWA) represents a valuable treatment for HCC at early stages and its potential impact on distant lesions in case of combined treatments has still to be investigated; Liao et al. examined this phenomenon in a mouse model with bilateral subcutaneous HCC nodules treated on the right side with various power levels of MWA with or without the association of ICIs. While MWA alone was observed to enhance distant tumor growth, the combination of high-power MWA with ICIs (anti-PD-1) increased the tumor infiltration by CD8+ T cells, reduced regulatory T cells, upregulated peripheral blood levels of TNF-α (Tumor Necrosis Factor-α), a T-helper1 (Th1)-type cytokine, and hindered distant tumor growth [41]; this peculiar immunological milieu favors and potentiates the abscopal effect [28,41].

Leuchte et al. examined the peripheral blood of a prospective cohort of 23 patients with HCC after a thermoablative treatment to rule out the tumor-specific immune response; they detected through fluorospot analyses that 30% of patients presented a de novo or increased specific response against the tumor, related to interferon-amma and interleukin 5. These subjects had a longer remission after MWA (more than one year) and a longer progression-free survival (PFS: 27.5 versus 10.0 months; *p* = 0.002). The following analysis of the patients who underwent combined treatment through MWA and resection, showed a disease-free survival of those subjects with a high abundance of T cells after MWA (37.4 versus 13.1 months; *p* = 0.03) [42]. This remarkable information stimulates further inquiry about the possible association of MWA with immunotherapy due to its immune-related effects. Duan et al. used a murine model (Hepa1-6 HCC) to confront MWA and ICIs (anti-PD-1/anti-CTLA-4) alone or in association; the results showed that combined treatment enhanced survival, reduced the recurrence rate, and potentiated both the infiltration of the tumor by cytotoxic T lymphocytes and the immune response by systemic T cells stimulated by MWA through the activation of the anti-neoplastic immunity. Moreover, the combined treatment (MWA plus ICIs) reduced Th2-type cytokines (with immune-suppressive characteristics), increased the cytokines by T-helper 1 cell (Th1-type) and stimulated the Th1-type immune response (with specific anti-neoplastic action) [43].

### 5.2. Radiotherapy

Radiotherapy (Table 1) is a helpful treatment for subjects who are not eligible for other treatments with radical therapeutic intent (surgery, chemoembolization, ablation) due to disease staging, to the precarious liver function, or being unable to face the risks related to specific procedures (e.g., administration of contrast medium, anesthesia, catheterization) [13,44].

There are several options for patients with HCC that are suitable for treatments based on radiotherapy: proton beam therapy (PBT), Trans Arterial Radioembolization (TARE), and SBRT. Previous radiotherapy techniques based on two- or three-dimensional techniques granted poor results in HCC due to hepatic risk of adverse events and functional decompensation. Modern radiotherapy is based on the modulation of the intensity of radiation dose (with progressive increase in the radiant dose), thus allowing one to reach the therapeutic success while preserving liver function [45,46]. Faced with “conventional” radiotherapy, SBRT allows one to optimize the therapeutic ratio, because it can apply an “ablative” radiant dose to the tumor, through short courses of large radiant doses for each radiotherapy fraction applied to small target volumes in order to obtain a consistent clinical response with very low and tolerable toxicity in normal tissues [47]. This ablative therapeutic scheme has a higher potential to trigger a strong systemic immune response against the neoplastic tissue than conventional radiotherapy [48].

**Table 1:** Pros and cons of radiotherapy depend on tumor volume, applied dose, and patient-related factors.

PROS [49]	Non-invasive method
	High local control of the tumor
	Delivered on an outpatient basis
	Organ preservation
	Preferred for patients with poor surgical candidates
	Chosen for tumors in anatomically challenging locations
	Symptoms palliation (pain, obstruction, bleeding)
CONS [50]	Toxicity (acute or chronic)
	Adverse events (fatigue, dermatitis, mucositis, fibrosis, organ disfunction)
	Risk of secondary malignant tumors
	Possible irradiation of healthy tissues
	Resource limitations

### 5.3. Immunotherapy

The recent introduction of immunotherapy in the landscape of HCC treatments increased the possibilities of survival for the patients with advanced HCC and allowed them to discover advantageous therapeutic effects in the case of combination with other treatments such as radiotherapy and increasing the interest around apparently unpredictable biological phenomena such as the abscopal effect; understanding how to direct the mechanisms that imply the abscopal effect represents an unmissable opportunity in enormously increasing the therapeutic success and expanding the survival possibilities in patients otherwise condemned to have a short span of life, despite treatment efforts [47].

ICIs have increasingly modified the management of advanced HCC, granting an increase in overall survival. They are frequently associated with the development of abscopal effects, even in tumors developed in organs different from the liver, when used in combination with local therapies such as ablation or radiotherapy. This strategy is encouraged by the Society of Interventional Radiology [13]. In fact, the association of ICIs with SBRT seems to be able to maximize the possibility of obtaining an abscopal effect on HCC [13,25].

The most common immunotherapy agents associated with the abscopal effect in advanced HCC are ICIs targeting the pathways involving PD-1/PD-L1 and CTLA-4:

- *Monotherapy with anti-PD-1 or anti-PD-L1*: There are several reports in the literature and some clinical trials about their involvement in abscopal effects after radiotherapy (atezolizumab, durvalumab, pembrolizumab, and nivolumab). A case report by Shu et al. stated that HCC developed on viral-related liver disease are more likely to present an abscopal effect after a treatment with SBRT and PD-1 inhibitor [20,51,52,53]. Chiang et al. reported a retrospective case series about five patients with advanced HCC undergoing SBRT followed by anti-PD1 (80% of these patients were treated also with a single chemoembolization (TACE) before SBRT). All the patients had an impressive response to the treatments (3/5 had partial response and 2/5 had complete response), did not develop progressive disease during the study follow-up (median 14.9 months), and their overall survival rate was 100%. Moreover, in one of the patients with partial response, it was possible to reduce the disease burden to a stage that allowed radiofrequency ablation. Due to the exiguous sample of patients, the authors suggest further trials to be held in order to deepen the knowledge of this phenomenon [54]. The phase 1–2 randomized clinical trial on 146 patients with unresectable HCC by Zhou et al. demonstrated that the ablation combined with toripalimab (anti-PD-1) improved PFS, compared with toripalimab alone (7.1 versus 3.8 months; HR 0.57; 95% CI 0.40–0.82; *p* < 0.001), alluding to a potential synergistic activity of the combination of local and systemic treatments [55].
- *Association of anti-PD-L1 (atezolizumab) and anti-VEGF (bevacizumab)*: It represents one of the first-line systemic treatments for HCC at an unresectable stage with increased survival and objective overall survival; this combination has well-recognized immunomodulatory properties along with anti-angiogenetic effects [36,56]. Nakabori et al. observed that, alongside the increase in immune response by the combination of atezolizumab and bevacizumab, SBRT is able to synergistically amplify such responses, even in subjects with an insufficient response to atezolizumab-bevacizumab when administered alone. In the case reported by Nakabori et al., a patient with metastatic HCC (secondary lesions in the lung and surrenal glands) was treated with atezolizumab plus bevacizumab followed by SBRT; pre-radiotherapy administration of ICIs did not reach a satisfying effect on disease control, that was obtained only after SBRT. Moreover, post-SBRT atezolizumab administered alone was not sufficient to trigger the abscopal effect, that was boosted by the addition of bevacizumab in the post-radiotherapy phase, reaching a complete disease response to treatments [57]. Yano et al. described the outstanding case of a patient with HCC and bone metastases, treated with atezolizumab and bevacizumab; despite the initial volume increase in all the neoplastic nodules and the worsening of the pain due to bone secondary localizations, after palliative radiotherapy of a metastatic lesion in the jaw, a noticeable shrinkage in tumor burden was observed, leading to ICIs discontinuation and complete response with absence of neoplastic recurrence at the following re-staging examinations [58].
- Dual blockade of the immune checkpoint:

(a) The association of durvalumab (anti-PD-L1) with tremelimumab (anti-CTLA-4) represents another first-line option (Single Tremelimumab Regular Interval Durvalumab “Stride” regimen) for the treatment of advanced HCC [7] and is gaining scientific interest for its possible implications in triggering the abscopal effect in synergy with radiotherapy. Kuwano et al. retrospectively analyzed 46 subjects with advanced HCC who underwent systemic treatment with tremelimumab plus durvalumab alone (23 patients) or in association with prior radiotherapy (16 patients); the latter presented a notably higher objective response rate compared with the ones treated only with ICIs without radiotherapy (56.3% versus 17.2%; *p* = 0.007) and a longer PFS (14.6 versus 2.7 months; *p* = 0.008), with better disease control rate (75.0% versus 34.4%; *p* = 0.008) and no significant differences in terms of adverse events. The biopsy performed after radiotherapy (before starting ICIs) presented higher intratumoral infiltration of CD8+ cells (87.5% versus 40.0%; *p* = 0.029), thus leading to an upregulation in the immune response inside the tumor microenvironment leading to the activation of the mechanisms underlying the abscopal effect, that may have contributed to such positive outcomes. The multivariate analysis underlined the role of prior radiotherapy as a significant predictor of an increased PFS (hazard ratio = 0.290, 95% CI = 0.106–0.794; *p* = 0.016) [59].
(b) The association of nivolumab (anti-PD-1) with ipilimumab (anti-CTLA-4) demonstrated a more efficient activation of systemic response after SBRT for the treatment of advanced HCC: Juloori et al. designed the first multicenter phase 1 prospective randomized trial studying the association of radiotherapy and ICIs, involving 14 patients treated with SBRT (40 Gy in five fractions) and subsequent nivolumab plus ipilimumab or nivolumab alone. The early closure of the study was due to slow accrual outcomes, but clinical outcomes (*p* < 0.05) were in favor of the group in which nivolumab and ipilimumab followed SBRT, compared with SBRT plus nivolumab alone, with an acceptable safety profile; respectively, overall response rates were 57% versus 0%, median OS 41.6 months versus 4.7 months, and median PFS 11.6 months versus 2.7 months [52].

No precise protocol has been established so far regarding the dosage and timing of ICIs administration or the specific treatment sequencing to enhance the abscopal effect. Each treatment follows its proper schedule: atezolizumab (1200 mg intravenous or 1875 mg subcutaneous) and bevacizumab 15 mg/kg (intravenous) every 21 days, tremelimumab (300 mg at the first administration) plus durvalumab (1500 mg every 28 days), ipilimumab 1 mg/kg (intravenous) every 42 days, and nivolumab 240 mg (intravenous) every 14 days [7,8,52,60].

**Figure 2:**
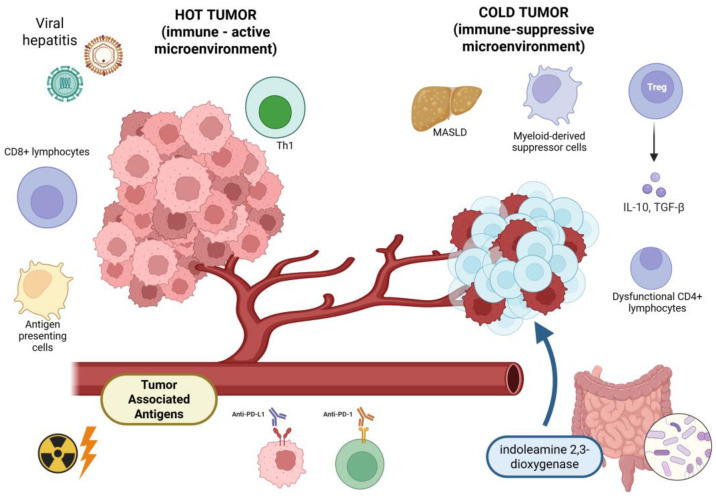
The abscopal effect, through the synergistic effect of radiotherapy and immune checkpoint inhibitors, can activate the tumor immune response, transforming a “cold” tumor into a “hot” one. Created with BioRender.com.

Through a murine model of HCC (Hepa1-6 mouse), Park JH et al. enquired into the mechanisms leading radiotherapy and anti-PD-L1 to functionally league and unleash the abscopal effect by overcoming the biological resistance to treatments of the neoplastic microcosm. The researchers found that the association of radiotherapy with systemic anti-PD-L1 can stimulate the anti-tumor immune response: increased levels of type 1 interferon, activation of dendritic cells in the lymph nodes draining from the tumor (through the amplification of the expression of PD-L1), and activation of cytotoxic CD8+ T cells in irradiated and non-irradiated tissues [61]. In fact, local radiotherapy determines the release of damaged molecules and antigens from the neoplastic tissues, enhancing the activation of the immune cells, but could collide against the ability of the tumor to adapt and develop resistance mechanisms that allow neoplastic cells to avoid damages provoked by the treatment. The association of a systemic therapy based on an anti-PD-L1 molecule acts synergically and increases the possibility to prevail over the neoplastic mechanisms of resistance, leading to a more efficient infiltration by T cells and subsequent control of the neoplastic disease by transforming an immune “cold” tumor (characterized by poor immune response) into a “hot” one (with a significant immune infiltration) and breaking through the neoplastic ability to evade immune response (Figure 2). These mechanisms represent the core of the abscopal effect [61,62]. An important highlight from the paper by Park et al. is the importance of the total dose and its distribution: the best results were obtained with a higher dose (5 Grays for 5 fractions) than a lower single dose (8 Gy for one fraction) [61].

The association of a systemic therapy based on an anti-PD-L1 molecule acts synergically and increases the possibility to prevail over the neoplastic mechanisms of resistance, leading to a more efficient infiltration by T cells and subsequent control of the neoplastic disease by transforming an immune “cold” tumor (characterized by poor immune response) into a “hot” one (with a significant immune infiltration) and breaking through the neoplastic ability to evade immune response. A possible strategy to trigger the transformation of a “cold” tumor into a “hot” one, is represented by oncolytic viruses, that are important immunotherapeutic agents in HCC and are being actively investigated. They can induce abscopal effect through immune-mediated mechanisms (direct oncolysis and intense activation of anti-neoplastic immune response), specifically when combined with other immunomodulatory treatments [63]. Recent clinical trials demonstrated that intratumoral administration of VG161 (an engineered herpes simplex virus expressing IL-12, IL-15, IL-15Rα, and a PD-1/PD-L1-blocking fusion protein) is able to mold HCC microenvironment provoking immune-activation even in “cold” tumors with previous resistance to systemic treatments. This characteristic can potentially determine the induction of abscopal effect by VG161 [63]. In fact, oncolytic viruses provoke cell death, releasing neoplastic neoantigens and increasing the activation of CD8+ T cells. These elements are at the core of systemic anti-neoplastic response. Preclinical models observed that synthetic oncolytic adenoviruses can reshape the HCC microenvironment and induce an abscopal effect in distant metastasis [64]. These preclinical findings suggest that the combination of oncolytic viruses with ICIs can improve local and distant anti-neoplastic response, thus triggering an abscopal effect [65].

## 6. The Safety of the Treatments Eliciting the Abscopal Effect

The aforementioned studies did not show an overall increase in adverse events in patients undergoing local treatments combined with ICIs. The main safety concerns in patients with HCC and underlying liver diseases, treated with combination therapies aimed at enhancing the abscopal effect, are immune-related hepatotoxicity, hepatic decompensation, and increased rates of treatment-related adverse events of grade ≥ 3. The risk and the severity of these events are affected by the type and severity of liver disease. No insurmountable safety concerns were identified in Child–Pugh A patients and in a selected group of Child–Pugh B [12,66]. Patients with decompensated cirrhosis (Child–Pugh score B or C) are at higher risk of both liver decompensation and developing adverse events and severe worsening of the hepatic decompensation when treated with locoregional therapies, ICIs, radiotherapy, or antiangiogenic agents, particularly in subjects with scarce baseline liver reserve [67].

The systematic review by Li et al. underlines the increase in treatment-related adverse events (even grade ≥ 3, with a relative risk of 1.25 [95% CI: 1.15–1.36]) in patients undergoing combined therapies, especially ICI (atezolizumab) plus anti-angiogenetic (bevacizumab) agents, compared with a single treatment alone. Moreover, they noticed that the incidence and severity of such adverse events is similar in Child–Pugh A and Child–Pugh B patients. The latter, despite experiencing an improvement in liver function after starting the therapy, have lower overall survival [68,69].

Severe immune-mediated liver damage (e.g., hepatic failure) is rare but can represent a heavy threat for the survival of patients with advanced liver cirrhosis [66].

The underlying liver disease and liver reserve are also an obstacle for the feasibility of the treatments for locally advanced or metastatic HCC due to the risk of functional decompensation or low tolerance to hepatotoxic therapies. The feasibility of these treatments is also influenced by performance status and comorbidities.

Even if radiotherapy schedules improved both safety profiles and tolerability, reducing the radiation-induced liver injury, it still requires an adequate patient selection.

Through a careful selection of the patients that are candidate to receive combined treatment, these risks can be minimized and, eventually, precociously managed.

## 7. Predictive Biomarkers for Abscopal Response in HCC

The factors that may predict prognosis in case of abscopal effect are involved in the interconnection between neoplastic biology, systemic inflammation, and immune response. There are still no validated, reliable HCC-specific biomarkers to predict response in case of abscopal effect; several biomarkers and immunological signatures have been associated with the response to abscopal effect determined by the combination of radiotherapy and ICIs in both HCC and other solid tumors, but none have been validated to enter daily clinical applications. Further research is mandatory to increase the predictivity of these biomarkers, thus offering new tools in the assessment of the HCC treatments outcome.

The mainly investigated potential biomarkers mirror the interplay between the different mechanisms that underlie the abscopal effect (activation of immune cells, neoplastic immunosuppressive activity, and systemic anti-tumor response):

- Neutrophil-to-lymphocyte ratio (NLR): The preclinical model by Mihaylov et al. suggests that lower NLR may predict abscopal responses when combined with CT/MRI radiomics features, but there is no clinical validation in HCC [70].
- Absolute lymphocyte count: The analysis by Chen et al. on three phase 1/2 clinical trials including patients with advanced solid tumors (among 153 subjects, only 5 had HCC) highlighted that higher absolute lymphocyte count after radiotherapy is related to strong activation of immune response, high probability of having an abscopal response, and better survival; despite a satisfying predictive value in preclinical studies, it has not yet been validated specifically for clinical practice [71,72].
- Peripheral blood cell signatures: Kästle et al. documented that baseline elevations in the platelet-to-lymphocyte ratio (PLR), neutrophil-to-lymphocyte ratio (NLR), and specific T cell and Natural Killer T cells could be associated with scarce response to local ablation therapy in HCC. Lower CD4+ T cell percentages, lower memory T cells, and decreased CD4/8 ratios, may prove reduced response, while higher PD-1+ CD4+ T cells may represent a favorable biomarker to be confirmed by future investigations [73].
- Extracellular vesicle proteomics: In advanced HCC, there are specific extracellular vesicle protein signatures that have been related to response to TARE and systemic treatment with sorafenib, but their concrete relevance to the abscopal response remains extremely uncertain [74].
- Tumor immune microenvironment: Several studies highlight a specific immunity signature as crucial in the pathogenesis of the abscopal effect (better outcomes to ICIs were documented with elevated levels of intratumoral CD8+ T cells, low ratio regulatory/effector T cells, signature of T-effector genes) [75].
- Effector tumor antigen-specific T cells: The quantitative determination of these cells could help in predicting the abscopal effect (more specifically after radiotherapy), because they mediate the response to anti-neoplastic treatments; these biomarkers are not yet available for clinical activity but are being studied in preclinical models, demonstrating promising results because they demonstrated a strong predictive value for abscopal effect [76].
- Cell-free DNA (cfDNA) and profiles of microRNA: Zafra et al. examined that the expression of specific microRNA in patients with lung cancer treated with stereotactic ablative radiotherapy and ICIs; they noticed that specific small RNAs (RNU2, SNORD1B, hsa-miR-1-3p and hsa-miR-133a-3p) could represent potential candidates as possible predictors of response to treatment. Patients with good responses to treatment had also a reduction in the concentration of circulating cfDNA (it has a strong predictive value in preclinical studies, but there is not yet validation in clinical practice) [77].
- Specific immune cells: Low levels of myeloid-derived suppressor cells are related to decreased immune-suppression by the tumor against the immune system, thus increasing the possibility of an abscopal effect [78]; increased levels of CD8+ T cells in non-irradiated lesions, distant from the irradiated ones, suggest a possible biomarker of an abscopal response [79].

At present, there is still little evidence about the importance of these biomarkers in predicting the abscopal effect and in facilitating the selection of patients undergoing specific treatments. Despite the satisfying results in some preclinical studies, they are not yet applicable in clinical practice. Future studies will probably highlight the role of each of these biomarkers, that are being tested in many different solid tumors as potential predictors of the abscopal effect, leading to the validation of some of them and the standardization of their application in clinical routine.

A recently published multicenter retrospective real-world cohort study by Trommer et al. [80] examined 142 patients with stage IV tumors treated with radiotherapy during or after the administration of ICIs: 61.3% had an abscopal effect and subsequently increased OS (18 versus 8 months; *p* < 0.01) and increased PFS (7 versus 3 months; *p* < 0.01). They also found that patient-related and timing-related factors were more predictive of abscopal effect than the details of radiotherapy treatment (e.g., radiation dose). No predictive value was achieved by tumor volume. Younger age and longer intervals between ICIs and radiotherapy were identified as possible predictors of abscopal events, while elevated C reactive protein (superior to 5 mg/L) and low body mass index (inferior to 25 kg/m^2^) are independent negative prognostic factors in terms of survival [16].

In the scientific literature, other positive prognostic factors are higher absolute lymphocyte levels both before and after radiotherapy (in case of combination with ICIs) [72], presence of high immunogenic tumors (with elevated antigen presentation) [16], increased levels of activated T cells, low populations of myeloid-derived suppressor cells, and elevated levels of tumor-specific antibodies (such as those against the “New York esophageal squamous cell carcinoma” NY-ESO-1) [73,79].

## 8. Conclusions

The abscopal effect belongs to the category of the “off-target” phenomena that may happen as a consequence of a treatment but, unlike others, it determines an advantage in the therapeutic panorama of several tumors as well as for HCC. Together with combined treatments, it represents an unvaluable therapeutic tool in the management of HCC, even if still “hidden” and almost unknown. It has all the prerogatives to become a powerful weapon that can be able to positively modify the disease prognosis. The possibility to boost the therapeutic response to a sequence of treatments could open important opportunities in the management of both localized and metastatic HCCs, proving alluring perspectives with both neoadjuvant and adjuvant therapeutic purposes.

Despite the considerable progress made in the treatment of advanced HCC, following the introduction of ICIs, objective overall survival remains modest and only a small group of patients achieve a real disease downstaging. The progressive deepening in the knowledge of the key molecular pathways underlying the abscopal effect may grant the possibility to manipulate the direction of the treatment and strengthen the outcome of HCC.

Another crucial point is represented by the understanding of the mechanisms involved in establishing HCC resistance to systemic treatments that are gaining continuously increasing interest and will doubtless represent a core topic in future research. If fully clarified, these mechanisms may help in directing the abscopal effect and open important changes in the clinical practice for the management of HCC, unlocking extraordinary therapeutic options even for patients now candidate only to palliative procedures.

Another critical challenge is constituted buy the absence of validated biomarkers that could help in the prediction of disease response with the aim of directing the clinical decision towards the most fitting combination of treatments, without losing sight of the underlying liver disease that can deeply interfere with a successful response not only through immunosuppressive mechanisms but also through the reduction in the patients’ tolerance to treatments, deeply related to liver disfunction and potentially interfering with the optimization of the sequence of combined treatments.

## Data Availability

No new data were created or analyzed in this study.

## Abbreviations

The following abbreviations are used in this manuscript:

AMP	Adenosine monophosphate
cfDNA	Cell-free DNA
CI	Confidence interval
CTLA-4	Cytotoxic T-Lymphocyte-Associated protein 4
DC	Dendritic cell
Gy	Grays
HCC	Hepatocellular carcinoma
ICIs	Immune checkpoint inhibitors
IL-6	Interleukin-6
MASH	Metabolic Dysfunction-Associated Steatohepatitis
MASLD	Metabolic dysfunction-associated steatotic liver disease
MWA	Microwave ablation
NLR	Neutrophil-to-lymphocyte ratio
PBT	Proton beam therapy
PD-1	Programmed cell death 1
PD-L1	Programmed cell death ligand 1
PFS	Progression-free survival
PLR	Platelet-to-lymphocyte ratio
ROS	Reactive oxygen species
SBRT	Stereotactic body radiotherapy
SIR	Society of Interventional Radiology
STING	Stimulator of interferon genes
TAAs	Tumor-associated antigens
TACE	Trans arterial chemoembolization
TARE	Trans arterial radioembolization
Th	T-helper
TKIs	Tyrosine kinase inhibitors (TKIs)
TNF-α	Tumor Necrosis Factor-α
VEGF A	Vascular endothelial growth factor A
